# Advanced glycation end products‐related modulation of cathepsin L and NF‐κB signalling effectors in retinal pigment epithelium lead to augmented response to TNFα

**DOI:** 10.1111/jcmm.13944

**Published:** 2018-10-19

**Authors:** Umar Sharif, Nur Musfirah Mahmud, Paul Kay, Yit C. Yang, Simon P. Harding, Ian Grierson, Tengku Ain Kamalden, Malcolm J. Jackson, Luminita Paraoan

**Affiliations:** ^1^ Department of Eye and Vision Science Institute of Ageing and Chronic Disease University of Liverpool Liverpool UK; ^2^ Eye Research Centre University of Malaya Kuala Lumpur Malaysia; ^3^ Ophthalmology The Royal Wolverhampton NHS Trust Wolverhampton UK; ^4^ Department of Musculoskeletal Science Institute of Ageing and Chronic Disease University of Liverpool Liverpool UK

**Keywords:** age‐related macular degeneration, cathepsin, NF‐κB signalling, inflammation, proteolysis, retinal pigment epithelium

## Abstract

The retinal pigment epithelium (RPE) plays a central role in neuroretinal homoeostasis throughout life. Altered proteolysis and inflammatory processes involving RPE contribute to the pathophysiology of age‐related macular degeneration (AMD), but the link between these remains elusive. We report for the first time the effect of advanced glycation end products (AGE)—known to accumulate on the ageing RPE's underlying Bruch's membrane in situ—on both key lysosomal cathepsins and NF‐κB signalling in RPE. Cathepsin L activity and NF‐κB effector levels decreased significantly following 2‐week AGE exposure. Chemical cathepsin L inhibition also decreased total p65 protein levels, indicating that AGE‐related change of NF‐κB effectors in RPE cells may be modulated by cathepsin L. However, upon TNFα stimulation, AGE‐exposed cells had significantly higher ratio of phospho‐p65(Ser536)/total p65 compared to non‐AGEd controls, with an even higher fold increase than in the presence of cathepsin L inhibition alone. Increased proportion of active p65 indicates an AGE‐related activation of NF‐κB signalling in a higher proportion of cells and/or an enhanced response to TNFα. Thus, NF‐κB signalling modulation in the AGEd environment, partially regulated via cathepsin L, is employed by RPE cells as a protective (para‐inflammatory) mechanism but renders them more responsive to pro‐inflammatory stimuli.

## 1 INTRODUCTION

The retinal pigmented epithelium (RPE) is a monolayer of highly specialized cells that underlie the neuroretina and help maintain retinal homoeostasis.^1^ Together with the underlying support matrix (Bruch's membrane, BrM), the RPE forms a selective barrier between the neuroretina and the choroid. In addition, the BrM is involved in modulation of RPE differentiation, migration and adhesion thus underpinning the role of the RPE–BrM complex in normal eye physiology.^2^, ^3^, ^4^

The longevity of RPE cells—owing to their terminally differentiated, non‐proliferative state^5^—makes them susceptible to numerous age‐related changes which in turn can impact on specialized cellular processes. The RPE undergoes several structural changes with age including loss of melanin, accumulation of lipofuscin and atrophy of RPE microvilli.^6^, ^7^, ^8^ The BrM also displays age‐related structural and physiological changes such as an increase in overall thickness and an increase in phospholipids, triglycerides and fatty acids content. In addition, collagen components of the BrM show increased cross‐linking and decreased solubility.^9^, ^10^, ^11^, ^12^ Gaining a better understanding of the ageing process and its impact on RPE cellular function has become a key area of research in age‐related macular degeneration (AMD), the most common cause of blindness in developed countries, whose pathophysiology is believed to be directly linked to RPE impairment.^13^, ^14^

An important phenomenon of ageing in all tissues is the accumulation of advanced glycation end products (AGE).^15^ AGE are a group of heterogeneous reaction products formed between reducing sugars and either lipids or the free amino groups on biomolecules such as proteins.^15^, ^16^ AGE accumulate on long‐lived extracellular matrix proteins such as collagen, where by altering macromolecular structure and function, they contribute to the development and progression of age‐related diseases.^16^, ^17^ AGE adducts are known to accumulate in the BrM with age and their presence has been associated with AMD.^18^, ^19^, ^20^ Exposure to AGE alters the gene expression profile of cultured RPE cells, which in turn impacts their functional capacity. It has been shown for example, that RPE cells grown on AGE‐modified substrate have been shown to have down‐regulated expression of cathepsins D, G and S.^19^

Cathepsins, such as the cysteine proteases cathepsins L and S and the aspartic protease cathepsin D govern lysosomal function.^21^, ^22^ Cathepsins B, D, L and S are known to be key regulators of autophagy.^23^, ^24^, ^25^ Within RPE cells, cathepsins D and S are involved in the degradation of photoreceptor outer segments (POS) and thus play a direct role in the maintenance of visual homoeostasis.^26^, ^27^ In addition, the activity of cathepsins has been linked to the modulation of signalling pathways; notably cathepsin L was shown to be involved in the regulation of the nuclear factor kappa B (NF‐κB) signalling pathway.^28^, ^29^ NF‐κB is a transcription factor that participates in the expression of many genes such as the pro‐inflammatory cytokines interleukin‐1β (IL‐1β) and interleukin 18 (IL‐18).^30^, ^31^ Both IL‐1β and IL‐18 are synthesized as precursors that require proteolytic maturation by caspase‐1 which must first be activated by multi‐protein complexes known as inflammasomes.^32^ As inflammation plays a major role in the pathogenesis of AMD,^33^ dysregulation of cathepsin activity might be a contributing factor to RPE dysfunction and AMD pathology.

Importantly in this context, cathepsins have been shown to be susceptible to age‐related alterations. An increase in cathepsin D activity along with a decrease in cathepsin L activity was documented in the ageing rat brain.^34^ The activity of cathepsins such as L and H significantly decreased in kidney proximal tubule cell line LLC‐PK1 after AGE exposure.^35^ A decrease in mRNA expression of lysosomal enzymes cathepsin S, cathepsin G, acid phosphatase, β‐galactosidase and β‐mannosidase was observed in RPE cells exposed to AGE; cathepsin D activity levels also decreased in RPE cells after AGE exposure.^19^ Moreover, in addition to cathepsins, AGE exposure was shown to modulate NF‐κB activity through activation of their receptor RAGE.^36^

Given the evidence that cathepsins can regulate NF‐κB activity, it is hypothesized that AGE adducts could exert their effects on the NF‐κB signalling pathway and thus on processes such as inflammation, through modulation of cathepsins activity. This study tested the above hypothesis in RPE cells, making use of an in vitro model of RPE cells exposed to AGE‐modified basement membrane mimicking an ageing BrM.^19^ Specifically, we analysed the effects of AGE on expression and activity of RPE‐expressed cathepsins alongside endogenous levels of effectors of the NF‐κB signalling pathway and investigated the link between cathepsin L and NF‐κB regulation. We demonstrate that following AGE exposure, both cathepsin L expression and activity, as well as protein levels of key NF‐κB pathway effectors, are reduced in RPE cells. Furthermore, we also show that cathepsin L is involved in regulation of NF‐κB regulation in RPE cells indicating the decrease of NF‐κB effectors following exposure to AGE may at least in part be because of changes in cathepsin L levels. We propose that the alterations of cathepsin L expression and activity and the associated dampening of the NF‐κB signalling serve as an early cellular protective mechanism in the ageing RPE, but may contribute to the environment in which cells are more vulnerable and receptive to subsequent or persistent pro‐inflammatory stimuli.

## 2 MATERIALS AND METHODS

### 2.1 RPE cell culture and AGE modification of extracellular matrix (ECM)

An authenticated human RPE cell line ARPE‐19 (ATCC, Rockville, Maryland, USA) was maintained in 1:1 mixture of DMEM/F12 (Sigma, Dorset, UK) media supplemented with 10% FCS for the first 4 days in culture after which the cells were maintained for long‐term culture in medium containing 2% FCS leading to the formation of stable RPE monolayers.

Experiments were carried out in standard 6‐well or 12‐well plates, previously coated with a solubilized basement membrane matrix extract, Matrigel (MG)™ (BD Biosciences, Oxford, UK) for 1 hour at 37°C. MG™, rich in common basement membrane matrix components, was used to mimic the innermost layer of the BrM. To mimic an aged phenotype of BrM, the Matrigel coat was AGE‐modified as previously described.^19^, ^37^ Briefly, AGE adduct formation was induced by incubating the MG™ substrate in the presence of 100 m mol L^−1^ glycolaldehyde (Sigma, Dorset, UK) at 37°C for 4 hours, followed by thorough washing with PBS. Termination of the glycation reaction was achieved by incubating the MG™ with 50 m mol L^−1^ sodium borohydride (Sigma, Dorset, UK) at 4°C overnight, followed by thorough washing. For control wells, MG™ was treated in the same way, with the exception of glycolaldehyde substitution with PBS. The degree of AGE modification and collagen cross‐linking in this ageing in vitro model was previously described.^37^ For the 6‐well plate experimental set‐up, ARPE‐19 cells were seeded on control and AGE‐modified MG™ at a cell density of 1 x 10^4^ cells per well; cell number was appropriately rescaled for 12‐well plate experiments.

### 2.2 Cathepsin L inhibition

ARPE‐19 cells were seeded at a density of 1 x 10^5^ on 6‐well plates and allowed to grow in culture for 4 days in DMEM/F12 (Sigma, UK) with 10% FCS to reach confluency. The confluent cells were treated with 40uM Cathepsin L Inhibitor III (Merck Millipore, Darmstadt, Germany) for 8 hours at 37°C and followed by thorough washing with PBS. Cells were lyzed in lysis buffer^38^ and subjected to SDS polyacrylamide electrophoresis for analysis of protein expression by Western blotting as described below.

### 2.3 TNFα treatment

Following the respective times for cathepsin L inhibition or AGE exposure, ARPE‐19 cells were treated with 10 ng/mL TNFα (ThermoFisher Scientific, Waltham, USA) for a further 2 hours. The control cells were not treated with TNFα. Cells were then thoroughly washed with PBS after which cell lysates were collected following the addition of lysis buffer^38^ to the wells. As the activation of NF‐κB signalling pathway in Hela cells in response to TNFα treatment is well documented,^39^ these cells were used as a positive control for TNFα activity and for the immunodetection of NF‐κB effectors.

### 2.4 Western immunoblotting

Protein content in cell lysates was determined using the Qubit fluorometer 2.0 (Invitrogen Ltd, Paisley, UK). Proteins in cell lysate samples were resolved by SDS‐PAGE, alongside a molecular weight marker (PageRuler Prestained Protein Ladder, Thermo Scientific, Rockford, USA) after which immunoblotting analysis was performed as previously described.^40^ To standardize and normalize across blots, an aliquot of a random sample was loaded on each gel as an internal control. Primary and secondary antibodies used are listed in Table 1. Protein detection was achieved using an enhanced chemiluminescent (ECL) substrate kit (Thermo Scientific, Rockford, USA) followed by imaging on the ChemiDoc BioRad ChemiDOC™ digital imager (BioRad, Hampstead, UK). Band densitometry values were obtained using Image Lab Software (Bio‐Rad, Hampstead, UK) and the readings were normalized against values of the internal control on each blot (given arbitrary value of 1) and to the loading control (glyceraldehyde 3‐phosphate dehydrogenase (GAPDH).

**Table 1:** Antibodies used for the analysis of protein expression levels

Antibody	Dilution
Anti‐cathepsin B (Abcam)	1:500
Anti‐cathepsin D (Abcam)	1:500
Anti‐cathepsin L (Abcam)	1:500
Anti‐cathepsin S (Abcam)	1:500
Anti‐NF‐κB p65 (Abcam)	1:500
Anti‐Phospho‐NF‐κB p65 Ser536P (Cell Signalling, Hertfordshire, UK)	1:500
Anti‐IkB‐α (Abcam)	1:500
Anti‐GAPDH (Abcam)	1:500
Secondary horseradish peroxidase (HRP)‐conjugated anti‐rabbit (Sigma‐Aldrich, Dorset, UK)	1:1000
Secondary horseradish peroxidase (HRP)‐conjugated anti‐rabbit (Sigma‐Aldrich)	1:2000

### 2.5 Real‐time quantitative PCR (qPCR)

RNA isolation was carried out using the RNeasy Plus Mini‐Kit (Qiagen, Hilden, Germany). Complementary DNA was synthesized from RNA using the First Strand cDNA Synthesis Kit (Thermo Scientific, Waltham, USA). Quantitative PCR was performed with the MESA BLUE qPCR Mastermix Plus Kit for SYBR assay (Low ROX; Eurogentec, Belgium) using a modified version of a previous protocol.^41^ Reactions were run on a Stratagene MX3000P qPCR System (Stratagene, California, USA), with a minimum of three biological replicates for each experimental condition and three technical replicates for each cDNA sample. Primer sets used are listed in Table 2. Final values were expressed relative to a calibrator sample assigned an arbitrary value of 1 and normalized to the expression of three housekeeping genes, beta tubulin, GAPDH and ribosomal protein L5 using the efficiency‐corrected ddCt method. The specificity of amplification reactions was confirmed by melt curve analysis.

**Table 2:** Primers used for gene expression level analysis

Cathepsin B	Forward	^5′^GCTTCGATGCACGGGAACAATG^3^
	Reverse	^5′^CATTGGTGTGGATGCAGATCCG^3′^
Cathepsin D	Forward	^5′^GCAAACTGCTGGACATCGCTTG^3′^
	Reverse	^5′^GCCATAGTGGATGTCAAACGAGG^3′^
Cathepsin L	Forward	^5′^GAAAGGCTACGTGACTCCTGTG^3′^
	Reverse	^5′^CCAGATTCTGCTCACTCAGTGAG^3^
Cathepsin S	Forward	^5′^TGGATCACCACTGGCATCTCTG^3′^
	Reverse	^5′^GCTCCAGGTTGTGAAGCATCAC^3′^
Beta tubulin	Forward	^5′^CTGGACCGCATCTCTGTGTACT^3′^
	Reverse	^5′^GCCAAAAGGACCTGAGCGAACA^3′^
GAPDH	Forward	^5′^TTGCCCTCAACGACCACTTT^3′^
	Reverse	^5′^TGGTCCAGGGGTCTTACTCC^3′^
Ribosomal protein L5	Forward	^5′^ATGCTCGGAAACGCTTGGT^3′^
	Reverse	^5′^GCGCAGACTATCATATCCCCC^3′^

### 2.6 Cathepsin enzyme activity assays

Enzymatic activities of cathepsins B, L, S and D were determined in ARPE‐19 exposed to AGE‐modified MG™ in parallel with ARPE‐19 cells exposed to control MG™ through the use of commercially available fluorometric based activity assays (Abcam, Cambridge, UK). All steps in this procedure were performed according to manufacturer's protocol with fluorescence measured in black 96‐well plates using the Fluostar Optima plate reader (BMG Labtech, Aylesbury, UK).

### 2.7 Statistical analysis

Data analysis was performed with commercial software Microsoft Excel (Version 2010, Microsoft UK Ltd, Reading, UK) and GraphPad Prism (Version 5, GraphPad Software, Inc., USA). A *P* value ≤0.05 was considered to be significant.

## 3 RESULTS

### 3.1 Cathepsins expression in RPE cells exposed to AGE: reduction of cathepsin L protein and activity levels

**Figure 1:**
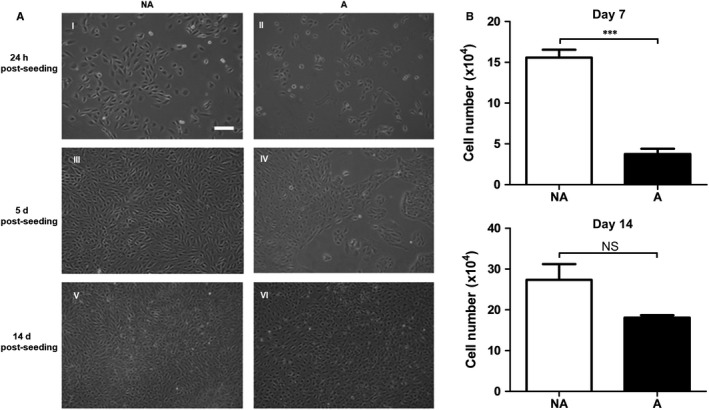
A, Morphology by phase contrast microscopy and growth characteristics of ARPE‐19 cells cultured on non‐modified MG™ (NA) (I, III and V) or AGE‐modified MG™ (A) (II, IV, VI). Representative image of cell cultures at 24 h post seeding (I, II), day 5 post seeding (III, IV) and day 14 post‐seeding (V, VI). Cells seeded on control NA MG™ presented a higher rate of growth and reached confluency quicker than the cells seeded on AGE‐modified MG™. Thus, at day 5 post‐seeding, ARPE‐19 cells had reached a confluent state when grown on control MG,™ whereas cells grown on AGE‐modified MG™ were ~40% confluent (III and IV). By day 14, ARPE‐19 cells grown on both control and AGE‐modified MG™ were confluent and had developed a cobblestone appearance (V and VI) making this time‐point appropriate for comparison studies. Scale bar represents 100 μm. B, Graph shows cell counts from ARPE‐19 cells grown on control NA MG™ and AGE‐modified MG™ for 7 and 14 d (average ± SEM, n = 3; Student's ***t*** **test**, ***P ≤ 0.001). At each time‐point, dead cells were washed away using PBS after which remaining cells were removed via trypsinization and counted using a haemocytometer. At 1 wk, (top graph) a significantly higher amount of cells were found on control NA MG™ compared to cells found on AGE‐modified MG™. By 2 wk (bottom graph), there was no significant difference between cell number on both control and AGE‐modified MG™

An in vitro system that mimics an important phenomenon of the ageing process, the accumulation of AGE, was used to determine the effects of ageing on the expression and activity of cathepsins in RPE cells. ARPE‐19 cells were cultured for 14 days on either untreated MG™ (NA) or AGE‐modified MG™ (A). Despite a slower rate of growth, RPE cells grown on AGE‐modified MG™ reached confluence and presented comparable epithelioid cell morphology by day 14 in culture (Figure 1A)—the time‐point thus chosen for experimental measurements. Furthermore, no significant difference in cell number between control and AGE‐modified MG™ at the 2‐week time‐point was observed (Figure 1B).

**Figure 2:**
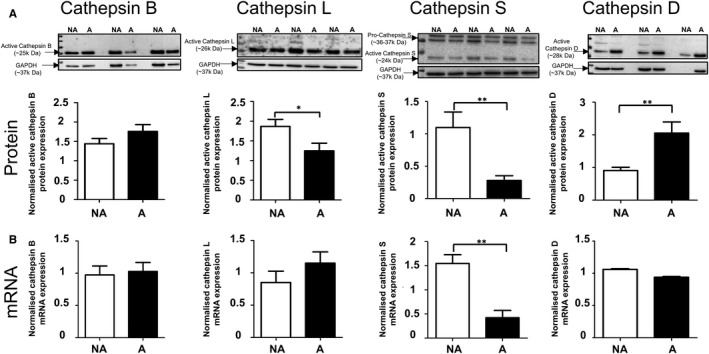
Analysis of expression levels of the cysteine proteinases cathepsins B, L, S and the aspartic proteinase cathepsin D in ARPE‐19 cells cultured on non‐modified MG™ (NA) and AGE‐modified MG™ (A) for 14 d. A, Protein levels of cathepsins B, L, S and D were assessed by immunoblotting. GAPDH immunodetection was used as a loading control and for normalization. Representative Western blots shown, with graphs presenting average normalized protein expression (active form; arbitrary units ± SEM, minimum of n = 9; Student's *t* test, *P ≤ 0.05; **P ≤ 0.01). Cathepsin L (active form) and cathepsin S (pro‐ and active forms) protein levels were significantly reduced in ARPE‐19 cells after AGE exposure. In addition, cathepsin D (active) levels were significantly up‐regulated in ARPE‐19 cells after AGE exposures. B, mRNA levels of cathepsins B, L, S and D were analysed by qRT‐PCR. Graphs show average expression normalized against three housekeeping genes as described in Methods (arbitrary units ±SEM, n = 3; Student's *t* test, **P ≤ 0.01). No significant changes were observed in mRNA levels for all cathepsins tested after AGE exposure with the exception of cathepsin S, which showed a significant decrease

Expression of cathepsins B, L, S and D was demostrated, both by immunoblotting and real time qPCR, in all ARPE‐19 cell lysates from these cultures (Figure 2). The analysis of expression showed that cathepsins L (active form) and S (pro‐ and active forms) protein levels were decreased in cells grown on AGE‐modified MG™ (Figure 2A). On the other hand, the aspartic protease cathepsin D (active form) showed an increase in protein levels in RPE cells grown on AGE‐modified MG™. The analysis of RNA levels showed no difference for all cathepsins tested in RPE cells grown on control MG™ vs AGE‐modified MG™, except for cathepsin S which displayed a decrease (Figure 2B). This observation indicates that protein alterations of cathepsins L and D are most likely because of post‐translational events whereas the decrease in total cathepsin S protein levels is a consequence of reduced transcription.

**Figure 3:**
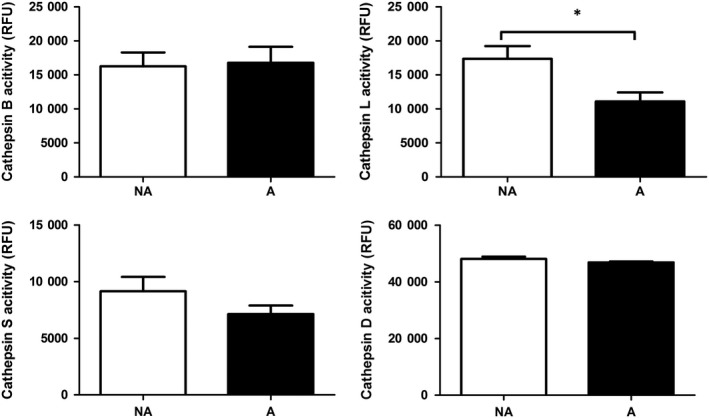
Activity analysis of cathepsins B, L, S and D in ARPE‐19 cells cultured on non‐modified MG™ (NA) and AGE‐modified MG™ (A) for 14 d. Activity levels were determined by fluorescence‐based activity assays. Cathepsin L activity was decreased in RPE cells after AGE exposure. Activity levels of cathepsins B, D and S remained unchanged in ARPE‐19 cells after AGE exposure. Graphs show average normalized activity in relative fluorescence units (RFU) (±SEM, minimum of n = 5; Student's *t* test, *P ≤ 0.05)

As the amount of protein is a factor that can affect cathepsin enzymatic activity, we thereafter analysed cathepsin‐linked enzymatic activity in RPE cells (Figure 3). When grown on AGE‐modified MG™, ARPE‐19 cells showed a significant decrease in cathepsin L‐linked enzymatic activity after 14 days in culture. All other cathepsins tested showed no significant changes of their activity level. Taken together, the data highlighted the significant down‐regulation of cathepsin L at both protein and importantly at activity level in ARPE‐19 cells cultured on AGE‐modified substrate.

### 3.2 Protein levels of key effectors of the NF‐κB pathway, p65, phospho‐p65 (Ser536) and IκBα are altered in RPE cells exposed to AGE

As cathepsin L is known to contribute to the regulation of NF‐κB signalling, alterations of this enzyme, as demonstrated by this study, could influence NF‐κB activity. Therefore, we investigated the effects of AGE on the regulation of NF‐κB regulation by first assessing the overall protein levels of total p65 and phospho‐p65 (Ser536), as well as the NF‐κB inhibitor IkBα in ARPE‐19 cells.

**Figure 4:**
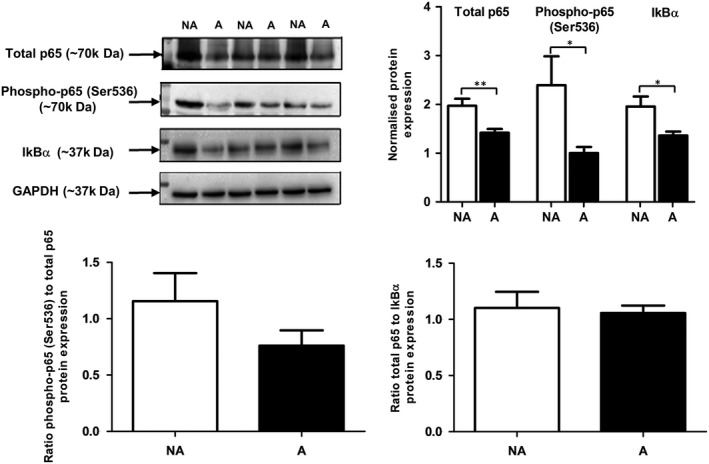
The effect of AGE on NF‐κB pathway effectors in ARPE‐19 cells. A, Protein levels of total p65, phospho‐p65 (Ser536) and IκBα in ARPE‐19 cells cultured on non‐modified MG™ (NA) and AGE‐modified MG™ (A) for 14 d were successively assessed by immunoblotting. GAPDH immunodetection was used as a loading control and for normalization. B, Graphs show average normalized protein expression (arbitrary units ±SEM, n = 10; Student's *t* test, *P ≤ 0.05; **P ≤ 0.01). Protein levels of total p65, phosph‐p65 (Ser536) and IkBα were all significantly decreased in ARPE‐19 cells after AGE exposure. C, Ratios of active phospho‐p65 (Ser536)/total p65 and total p65/IkBα showed no significant difference between non‐AGE and AGE conditions, indicating decrease of respective protein levels at similar rates

A significant decrease of both total p65 and phospho‐p65 (Ser536) protein levels (consistent with decreased mRNA levels for p65, data not shown) was observed in RPE cells exposed to AGE‐treated Matrigel, compared with control cells cultured in non‐AGEd conditions (Figure 4A,B). IkBα protein levels were also decreased in RPE cells exposed to AGE, suggesting an overall decrease in the NF‐κB signalling pathway in RPE cells exposed to AGE. Furthermore, the ratios of phospho‐p65 (Ser536P)/total p65 and total p65/IkBα protein levels showed no significant alterations between cells exposed to AGE and control cells (Figure 4C‐D). The results indicated a similar decrease in protein and activity levels of these key effectors of the NF‐κB signalling pathway subsequent to exposure to the AGEd environment, suggestive of an AGE‐related cellular response resulting in dampening of this signalling pathway.

### 3.3 Effect of cathepsin L inhibition on the constitutive expression of NF‐κB signalling effectors in RPE cells

**Figure 5:**
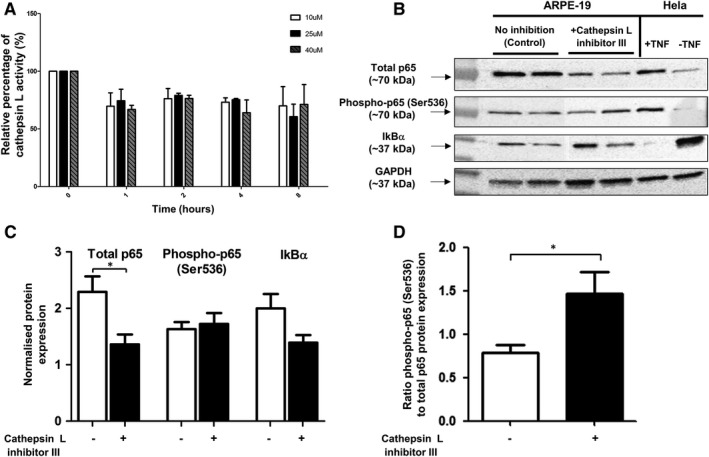
The effect of cathepsin L activity inhibition on the expression of NF‐κB signalling effectors in RPE cells. A, Evaluation of cathepsin L inhibitor III concentration and exposure time for effective enzymatic activity inhibition in ARPE‐19 cells. Significant decrease of the enzymatic activity observed up to 8 h post‐treatment at inhibitor concentrations of 25 μmol L^−1^ and 40 μmol L^−1^. B, Immunoblotting analysis of total p65, phospho‐p65 (Ser536) and IkBα protein levels in ARPE‐19 cells, untreated and treated with 40 μ mol L^−1^ cathepsin L inhibitor III. HeLa cells ± TNFα were used as controls. GAPDH immunodetection was used as a loading control and for normalization. C, Comparison of protein expression of total p65, phospho‐p65 (Ser536) and IkBα normalized to GAPDH level in the absence and presence of cathepsin L inhibition (arbitrary units ±SEM, minimum of n = 10; Student's *t* test, *P ≤ 0.05). Protein level of total p65 was significantly decreased in ARPE‐19 cells in the presence of cathepsin L inhibition (D) Ratio of phospho‐p65 (Ser536)/total p65, indicating the proportion of activated p65 in the total p65 protein pool, in the absence and presence of cathepsin L inhibition (minimum of n = 10; Student's *t* test, *P ≤ 0.05). Significant increase in ratio was observed in ARPE‐19 cells in the presence of cathepsin L inhibition compared to control cells. This indicates a higher amount of activated p65 from the total p65 protein pool

In order to investigate the potential functional link between cathepsin L and regulation of NF‐κB signalling in RPE cells, the protein levels of total p65, phospho‐p65 (Ser536) and IkBα were measured and compared in the presence and absence of the irreversible cathepsin L inhibitor III (Merck Millipore). Optimization of inhibitor concentration/time course experiments were carried out and showed that concentrations of 25 μ mol L^−1^ and 40 μ mol L^−1^ of cathepsin L inhibitor were sufficient to inhibit cathepsin L activity for up to 8 hours in RPE cells, with viability unaffected in all conditions. We therefore used the highest concentration (40 μ mol L^−1^) and the longest time‐point (8 hours) for subsequent experiments to ensure effective and sustained cathepsin L inhibition (Figure 5A). A significant decrease of total p65 protein level was observed in ARPE‐19 cells treated with cathepsin L inhibitor III, consistent with a role for cathepsin L in modulation of NF‐κB signalling (Figure 5B,C). Protein levels of phospho‐p65 (Ser536) and IkBα were not significantly altered (although the latter slightly decreased). Thus, the overall outcome of the cathepsin L inhibition translated into a significant increase of the phospho‐p65 (Ser536)/total p65 ratio (Figure 5D). Taken together, these results indicate that the overall decrease of the total p65 protein pool upon cathepsin L inhibition is accompanied by the enhancement of the proportion of activated p65 (Ser536) in the total p65 cellular pool, thus potentially shifting the profile of p65 activity, similar to signal priming events.

### 3.4 Effect of cathepsin L inhibition on the TNFα‐induced NF‐κB signalling in RPE cells

**Figure 6:**
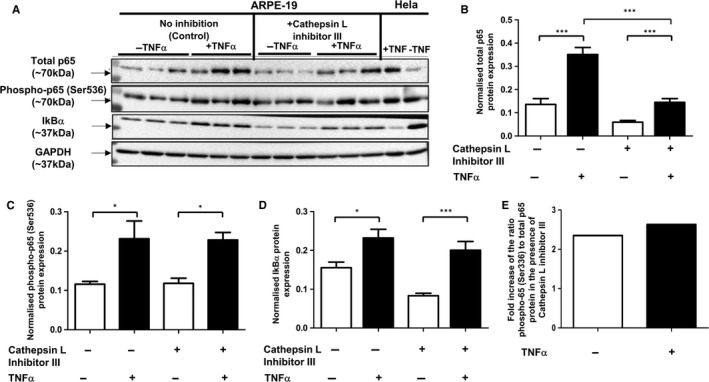
The effect of TNFα treatment on the level of NF‐κB signalling effectors in RPE cells after cathepsin L inhibition. A, total p65, phospho‐p65 (Ser536) and IkBα protein expression determined by Western blotting analysis in ARPE‐19 cells exposed to ± cathepsin L inhibition and ± TNFα treatment. (B‐D) Graphs show average protein expression normalized to GAPDH (arbitrary units ±SEM, minimum of n = 8; One way ANOVA followed by Tukey's multiple comparison test, *P ≤ 0.05; ***P ≤ 0.001). E, Ratios of phospho‐p65 (Ser536)/total p65 indicate the proportion of phosphorylated p65 (and thus potentially active) in the total p65 pool in RPE cells; data demonstrates similar fold increase of these ratios upon TNFα stimulation and cathepsin L inhibition

After determining that cathepsin L activity contributes to modulation of p65 protein levels in RPE cells, we next investigated whether the NF‐κB signalling pathway response to the pro‐inflammatory stimulus TNFα is altered following inhibition of cathepsin L activity. In both control (without cathepsin L inhibition) and treated (with cathepsin L inhibitor III) ARPE‐19 cells, a significant increase of total p65, phospho‐p65 (Ser536) and IkBα protein levels were observed after TNFα exposure (Figure 6A‐D). Importantly, however, there was no significant difference between the fold increase of the ratio of phospho‐p65 (Ser536)/total p65 induced by TNFα and by cathepsin inhibition alone (Figure 6E). These data also corroborated the effect of cathepsin L inhibition on the profile of active vs total p65 pool demonstrated for unstimulated conditions (Figure 5D).

### 3.5 Effect of AGE on the TNFα‐induced NF‐κB signalling effectors in RPE cells

**Figure 7:**
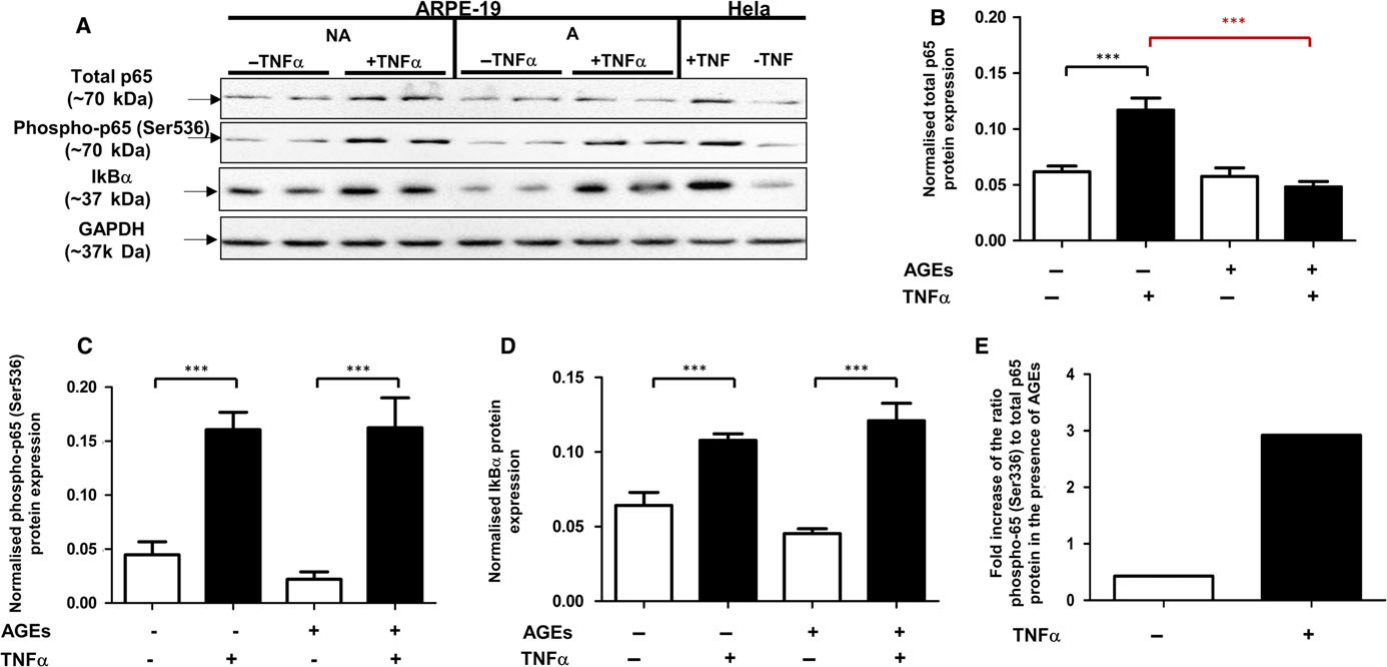
The effect of TNFα treatment on the response of NF‐κB signalling effectors in RPE cells after AGE exposure. A, Total p65, phospho‐p65 (Ser536) and IkBα protein expression determined, alongside normalizing GAPDH, by Western blotting analysis of ARPE‐19 cells cultured on non‐modified MG™ (NA) and AGE‐modified MG™ for 14 d. (B‐D) Average normalized total p65, phospho‐p65 (Ser536) and IkBα protein expression, respectively (arbitrary units ± SEM, minimum of n = 8; One way ANOVA followed by Tukey's multiple comparison test, ***P ≤ 0.001). E, Fold increase of the ratio phospho‐p65 (Ser536)/total p65 following stimulation with TNFα in cells is augmented in AGE‐exposed cells

After observing that cathepsin L activity modulates the level of NF‐κB signalling effectors in RPE cells, we sought to determine the response to TNFα in AGE‐exposed RPE cells, where cathepsin L activity is decreased. TNFα treatment led to a significant increase in levels of total p65 only in control cells with overall levels remaining unaffected in AGE‐exposed cells (Figure 7A,B). Phospho‐p65 (Ser536) and IkBα were significantly increased in both control and AGE‐exposed cells after exposure to TNFα (Figure 7C,D).

As AGE can independently influence the different NF‐κB effectors, the actual effect of TNFα on the NF‐κB signalling response is best represented by the phospho‐p65 (Ser536)/total p65 ratio. Thus, although the ratio of phospho‐p65 (Ser536)/total p65 was significantly increased for both control and AGE‐exposed cells after TNFα treatment (indicating a functional NF‐κB signalling pathway), this ratio was significantly higher in RPE cells exposed to AGE, indicating a higher proportion of active p65 in the general pool in an AGE‐containing environment. This is illustrated by the substantial (approximately six times higher) fold increase of the ratio of phospho‐p65 (Ser536)/total p65 induced by TNFα in the presence of AGE (Figure 7E). Overall the data show an increased activation of the NF‐κB signalling in a higher proportion of cells and/or through an enhanced response to TNFα when cells are exposed to AGE.

## 4 DISCUSSION

In this study, we demonstrated that cathepsin L expression and its enzymatic activity as well as key NF‐κB signalling pathway effectors decrease in RPE cells exposed to AGE. In addition, we showed that cathepsin L is involved in modulating the NF‐κB pathway, indicating that AGE‐induced alterations of NF‐κB effectors may, at least in part, be a consequence of the changes in cathepsin L levels. Unexpectedly, the AGE‐related constitutive dampening of the NF‐κB effectors created an environment in which cells mounted an increased response to TNFα, indicating that the cells exposed to AGE are more sensitive and more responsive to pro‐inflammatory conditions. This data highlights possible key mechanisms in which alterations to cathepsin L and NF‐κB play a role in the physiology of the ageing RPE.

The cellular model used in this study exploited the presence of AGE in the ECM of RPE cells to mimic one aspect of the ageing process of these cells. Glycolaldehyde was used to induce AGE formation on the basement membrane matrix as glycolaldehyde‐derived AGE adducts involving reactive α‐oxaloaldehydes have been observed in human BrM.^19^, ^42^ The use of glycolaldehyde to induce AGE formation on matrix in vitro, along with the degree of AGE adduct formation and crosslinking have been described previously.^19^, ^37^, ^42^ A progressive rise in AGE has been observed in the BrM with age.^18^, ^19^ As a direct relationship between the RPE and BrM exists, AGE deposition on the BrM may be partially attributed to RPE dysfunction and linked to subsequent atrophy and photoreceptor degeneration.

We report here the characterization of expression of cathepsins B, H, L and D in ARPE‐19 cells and the changes in their expression in cells exposed to AGE. The most prominent change induced by the presence of AGE was for cathepsin L, which showed significantly decreased protein and activity levels (Figures 2 and 3). Being a potent lysosomal protease, the implications of decreased cathepsin L activity could have severe impact on crucial proteolysis‐related RPE functions such as POS degradation and autophagy, processes which when impaired contribute to the accumulation of cellular debris such as lipofuscin and ultimately lead to cellular dysfunction.^19^, ^43^, ^44^ Notably in the context of RPE function, in addition to core lysosomal functions, cathepsin L was previously shown to be involved in complement and NF‐κB activity regulation.^28^, ^29^, ^45^, ^46^

Dysregulated complement plays a key role in AMD development.^47^ Interestingly, survival of immune cells was shown to be dependent on intracellular cathepsin L‐mediated cleavage of the key complement component C3 into biologically active C3a and C3b fragments.^45^ In addition, it was also demonstrated that complement factor H (CFH), an inhibitor of the complement pathway, binds to apoptotic RPE cells and is internalized where it acts as a co‐factor enhancing cathepsin L‐mediated cleavage of C3 and opsonization.^46^ This then aids removal of damaged/dysfunctional RPE cells as well as preventing excessive inflammation. As cathepsin L‐mediated cleavage of C3 contributes to survival of cells, it is possible that a reduction of this enzyme in RPE cells exposed to AGE, a known inducer of cell death,^48^ contributes to a cellular environment unfavourable for RPE survival. Furthermore, damaged/dysfunctional RPE cells would not efficiently be removed because of reduction in cathepsin L‐mediated cleavage of C3 and subsequent diminished opsonization. An accumulation of dysfunctional cells may then contribute to biogenesis of material such as drusen and further exacerbate inflammatory conditions.

Of particular interest and a focus in this study was the role of cathepsin L in NF‐κB activity regulation. Cathepsin L was shown to have a dual role in NF‐κB regulation, being involved in the activation as well as the suppression of NF‐κB activity.^28^, ^29^ Current evidence supports the idea that increased activation of NF‐κB, as a key transcriptional regulator of genes involved in processes such as inflammation and apoptosis,^30^, ^31^, ^49^, ^50^ is a driving force behind the ageing process.^51^ It is also known that AGE exposure leads to NF‐κB activation.^36^ As cathepsin L can regulate NF‐κB activity, it is possible that AGE exert their effects on the NF‐κB signalling pathway, at least in part, through modulation of cathepsin L levels.

To test the above hypothesis, we investigated the impact of AGE on levels of key NF‐κB effectors—p65, phospho‐p65 (Ser536) and IkBα, and addressed the role of cathepsin L in NF‐κB regulation in RPE cells through the use of a chemical activity inhibitor. Surprisingly, following exposure to AGE, RPE cells displayed decreased total p65, phospho‐p65 (Ser536) form and IkBα protein levels, which suggested an overall decrease in the NF‐κB signalling system (Figure 4). Cathepsin L inhibition led to decreased protein level of total p65 in RPE cells which suggested that the decrease in cathepsin L activity may contribute to the decrease of total p65 seen in AGE‐exposed RPE cells. Notably, however, the overall decrease of the total p65 protein pool upon cathepsin L inhibition was accompanied by the enhancement of the proportion of activated p65 (P‐Ser536) in the total p65 cellular pool, thus indicating a shift in the profile of p65 activity.

Alterations in NF‐κB signalling effectors lead to changes in genes and processes regulated by this pathway. We know that p65 regulates the expression of pro‐apoptotic genes such as p53, a tumour suppressor that induces cell death.^49^, ^50^ In previous studies, AGE exposure (up to 48 hours) of RPE cells was shown to induce cell death, a response associated with increased oxidative insult.^48^ In the 2‐week AGE‐exposed RPE cell culture system investigated in our study, it was observed that AGE‐exposed cells had a slower rate of growth but did reach confluence and comparable morphology to control cells by day 14 (Figure 1). This slower rate of growth could be explained by the known AGE‐induced impairment of replicative capacity.^37^ Furthermore, it is possible that once cells were seeded onto the AGE‐modified basement membrane, a level of cell death occurred in the first few days which led to a decrease in cell number and thus a lag behind controls in reaching confluency. Cells that manage to survive on the AGE‐modified substrate initiated mechanisms and adapted to their environment enabling them to remain viable. Decreased NF‐κB activation following cathepsin L inhibition was linked functionally with protection against apoptosis.^28^ Thus, dampening of the NF‐κB signalling pathway, which may at least in part be because of decreased cathepsin L levels in RPE cells, could be a protective mechanism that helps remaining cells on the AGE‐modified basement membrane maintain viability in spite of the adverse and damaging effects of AGE.

Down‐regulation of NF‐κB activity can also influence the expression of inflammatory genes such as IL‐1β and IL‐18 and affect inflammation processes.^30^, ^31^ Clarification of how inflammation arises and is modulated in the ageing RPE is crucial for understanding how AMD develops. AGE exposure leading to decreased NF‐κB activity may indicate that RPE cells are able to mount an initial protective response against inflammatory stimuli. The idea of cells protecting themselves from damaging stimuli is in line with the concept of “para‐inflammation,” an adaptive response to cellular/tissue malfunction which aims to maintain sufficient functionality.^52^ However, if cell/tissue stress persists and is not removed, then cells are tipped from a para‐ to a chronic inflammatory state.^52^ Para‐inflammation was described in the ageing retina as a stress response aimed at maintaining tissue integrity which is lost during chronic inflammation, contributing to the development of AMD.^53^ Our data highlights the shifting in expression patterns of NF‐κB effectors as key mediators of inflammation in RPE cells in response to the age‐related ubiquitous factor, AGE. Interestingly, a recent study showed that RPE cells exposed to AGE for 24 hours displayed up‐regulation and down‐regulation of different pro‐ and anti‐inflammatory cytokines.^54^ This complex pattern of secretion was said to reflect a “para‐inflammatory” response of RPE cells after 1‐day exposure.^54^

In the present study RPE cells were exposed to AGE for 2 weeks in order to create a more “chronic” exposure. However, the dampening of the NF‐κB pathway at this time‐point may still be reflective of a “para‐inflammatory” survival response as the in vitro model used is likely to reflect the initial adaptive response of RPE cells to the presence of AGE. The RPE cells in situ undergo a slow progression of insult by experiencing cumulative age‐related changes and damage. It is conceivable that the RPE cells use the para‐inflammatory response as an initial protective mechanism, but may eventually succumb to prolonged or enhanced damage associated with a chronic condition. It should also be pointed out that although traditionally seen as a pro‐inflammatory mediator, NF‐κB can also regulate anti‐inflammatory genes which add extra complexity to this signalling pathway.^55^

From a para‐inflammation state, cells can be tipped in a direction that overwhelms cellular defences via constant or additional stresses to cause dysfunction. We therefore also investigated the response to the pro‐inflammatory stimulus TNFα of RPE cells that had been cathepsin L‐inhibited or AGE exposed. In addition to being an inducer of the NF‐κB pathway, TNFα presents increased expression with human ageing and in age‐related degenerative diseases such as Alzheimer's disease.^56^ Importantly in relation to the RPE, TNFα was shown to increase the production and secretion of the angiogenic VEGF protein, a known contributor to development of wet (neovascular) AMD,^57^ and anti‐TNFα injections helped improve vision of wet AMD patients.^58^

Our data provides experimental evidence that in cells exposed to AGE, the phospho‐p65 (Ser536)/total NF‐κB p65 ratio is significantly higher compared to non‐AGE cells when treated with TNFα. This is particularly important functionally, as the higher proportion of active p65 in the total cellular pool in an AGE‐exposed environment translated into a higher fold increase of the ratio of phospho‐p65 (Ser536)/total p65 induced by TNF‐α in the presence of AGE compared to the ratio in the presence of AGE alone (Figure 7E), hence revealing that “aged” RPE cells mount an increased response to pro‐inflammatory stimuli. Interestingly, no significant difference between fold increase of the ratio of phospho‐p65 (Ser536)/total p65 was observed in cells treated with TNF‐α and cathepsin L inhibition alone (Figure 6E). This shows that cathepsin L inhibition, which only seems to influence total p65 levels, is not sufficient to make cells more responsive to pro‐inflammatory stimuli on its own.

In conclusion, our data indicate that the presence of AGE adducts, a characteristic of the ageing process, renders RPE cells more responsive to pro‐inflammatory stimuli and that cells become more vulnerable and responsive to an inflammatory stimulus in an “aged” environment. This may not be an RPE‐specific response because TNFα−induced apoptosis is also enhanced in T cells from elderly patients compared to young ones.^59^ Also, bone marrow‐derived macrophage from aged rats were more responsive to pro‐inflammatory stimuli compared to young macrophage.^60^ Thus, collectively data from different types of cells indicate that age‐related processes, of which AGE accumulation is just one, directly affect the cellular response to inflammatory stimuli.

## CONFLICT OF INTERESTS

The authors have no competing interests.

## AUTHOR CONTRIBUTIONS

LP, TAK and MJ conceived and designed the study and analysed the results. US, NMM and PK performed the experiments and carried out the data analysis. US, NMM, PK and LP wrote the paper. All authors were involved in editing the manuscript or revising it critically. All authors read and approved the final manuscript.
